# Alpha-Synuclein Levels in Blood Plasma Decline with Healthy Aging

**DOI:** 10.1371/journal.pone.0123444

**Published:** 2015-04-06

**Authors:** Niklas K. U. Koehler, Elke Stransky, Mirjam Meyer, Susanne Gaertner, Mona Shing, Martina Schnaidt, Maria S. Celej, Thomas M. Jovin, Thomas Leyhe, Christoph Laske, Anil Batra, Gerhard Buchkremer, Andreas J. Fallgatter, Dorothee Wernet, Elke Richartz-Salzburger

**Affiliations:** 1 Department of Psychiatry and Psychotherapy, Eberhard-Karls-University Tübingen, Calwerstr. 14, 72076 Tübingen, Germany; 2 German Center for Neurodegenerative Diseases (DZNE), Otfried-Müller-Strasse 27, 72076 Tübingen, Germany; 3 Zentrum für Klinische Transfusionsmedizin, Otfried-Müller-Strasse 4, 72076 Tübingen, Germany; 4 Center of Old Age Psychiatry, Psychiatric University Hospital, Wilhelm Klein-Strasse 27, CH-4012 Basel, Switzerland; 5 Laboratory for Cellular Dynamics, Max-Planck-Institute for Biophysical Chemistry, Am Faßberg 11, 37077 Göttingen, Germany; 6 Department of Biological Chemistry (CIQUIBIC, CONICET), School of Chemical Sciences, National University of Córdoba, Haya de la Torrey Medina Allende, Ciudad Universitaria, X5000HUA Córdoba, Argentina; UCL Institute of Neurology, UNITED KINGDOM

## Abstract

There is unequivocal evidence that alpha-synuclein plays a pivotal pathophysiological role in neurodegenerative diseases, and in particular in synucleinopathies. These disorders present with a variable extent of cognitive impairment and alpha-synuclein is being explored as a biomarker in CSF, blood serum and plasma. Considering key events of aging that include proteostasis, alpha-synuclein may not only be useful as a marker for differential diagnosis but also for aging per se. To explore this hypothesis, we developed a highly specific ELISA to measure alpha-synuclein. In healthy males plasma alpha-synuclein levels correlated strongly with age, revealing much lower concentrations in older (avg. 58.1 years) compared to younger (avg. 27.6 years) individuals. This difference between the age groups was enhanced after acidification of the plasmas (p<0.0001), possibly reflecting a decrease of alpha-synuclein-antibody complexes or chaperone activity in older individuals. Our results support the concept that alpha-synuclein homeostasis may be impaired early on, possibly due to disturbance of the proteostasis network, a key component of healthy aging. Thus, alpha-synuclein may be a novel biomarker of aging, a factor that should be considered when analyzing its presence in biological specimens.

## Introduction

Alpha-synuclein, an aggregation-prone and amyloid-forming protein, plays a pivotal role in the pathogenesis of synucleinopathies such as Parkinson’s disease (PD), dementia with Lewy bodies (DLB) and multiple system atrophy (MSA) and is being explored as a biomarker in cerebrospinal fluid (CSF), blood plasma, and serum [1,2]. Recently, the aggregation of disease-related proteins in physiological aging has been attributed to altered protein homeostasis (proteostasis) [3,4]. This was suggested to represent a biomarker of aging that could modulate life span and cause neurodegeneration [5,6]. Identification and application of such biomarkers may advance longevity and help influence or prevent concomitant illnesses. Other markers of aging or frailty are related to the immune system, such as innate or adaptive immune cells (immunosenescence) [7,8], cytokines [9], inflammation and autoimmunity [10], and genetic or epigenetic signatures [11], such as telomere length or DNA methylation state, respectively.

Alpha-synuclein is one of two other amyloidogenic key proteins, specifically, amyloid beta (Abeta) and tau, which play a major pathophysiological role in the three most common neurodegenerative diseases leading to dementia, namely Alzheimer’s disease (AD), DLB and Parkinson’s disease dementia (PDD). The three proteins also appear to act in concert by co-aggregation [12–16] and propagation through the nervous system tissue in a prion-like manner leading to neuronal death [14,17,18].

Reliable, high throughput and cost-effective measurement of alpha-synuclein in biological fluids is of crucial importance for clinical research. However, published concentrations in biological samples with high matrix effects such as plasma or serum vary widely. We developed a highly specific and validated ELISA for the measurement of total alpha-synuclein and analyzed plasma from healthy males (n = 80) in a younger (range 22–35 years) and older age group (50–69 years). Plasma from the same specimens, acidified to dissociate alpha-synuclein-protein complexes, was analyzed in parallel. Our findings may impinge on studies of the pathophysiological mechanisms of aging involving impaired proteostasis and have implications for the preclinical diagnosis of neurodegenerative diseases.

## Materials and Methods

### Plasma samples

The ethics committee of the Eberhard-Karls-University Tübingen approved this study (ethics nr. 314/06). Eighty blood plasmas from healthy male blood donors and 13 cell free CSF samples from individuals recruited by the Department of Transfusion Medicine and Department of Psychiatry and Psychotherapy, Eberhard-Karls-University Tübingen were analyzed. Written informed consent was received by all individuals participating in the study. If capacity to consent of participants was compromised by any means, based on neuropsychological testing and physicians’ judgment, legal guardians consented on their behalf. All participants who declined to participate or otherwise did not participate in the study were not disadvantaged in any other way. Individuals did not suffer from a physical or brain disorder and were unremarkable in cognition.

Blood and/or CSF were taken between 9 and 12 a.m., processed within 2 hours (h) after collection, aliquoted and immediately frozen at -20°C. In detail, blood was drawn into polypropylene (pp) lithium-heparin tubes (Sarstedt-S-Monovette, Germany). CSF was collected from the lumbar cistern (between lumbar vertebrae L2-L5) though a 22 G spinal needle (Becton, Dickinson and Company, USA) into a 13 ml pp tube (Sarstedt). After centrifugation at 2000xg for 15 minutes at 4°C, cell-free plasma and CSF were aliquoted into polypropylene Eppendorf microcentrifuge tubes (Eppendorf, Hamburg, Germany) or Nunc Cryo tubes (Thermo Fisher Scientific, MA, USA).

### Data and statistical analysis

Statistical evaluation and regression analysis were carried out as indicated using the GraphPad Prism 5 software (La Jolla, CA, USA). Groups were compared by unpaired or paired two-tailed t-test, as indicated. Demographics were analyzed by unpaired two-tailed t-test. Correlations were assessed by Spearman correlation. The significance level was set at p<0.05 (***p<0.001, highly significant; **p = 0.001–0.01, very significant; *p = 0.01–0.05, significant; >0.05 not significant).

### Measurement of alpha-synuclein concentration

Human alpha-synuclein was measured by a highly specific and sensitive sandwich enzyme-linked immunosorbent assay (ELISA) developed in our laboratory and described below. Human recombinant alpha-synuclein produced in *E*. *coli* was analyzed for purity by western immunoblot and used as a positive control and to generate a standard curve. Ninety six well plates (Nunc Maxisorb, Thermo Fisher Scientific, MA, USA) were coated with 3 μg/ml monoclonal mouse anti-alpha-synuclein IgG1 kappa, clone syn211 (Sigma-Aldrich, Deisenhofen, Germany) in carbonate-bicarbonate buffer at pH 9,6 and incubated overnight at 4°C. Plates were washed in PBS/Tween (PBS, Sigma-Aldrich, 0.1% Tween-20) and blocked in TBS/Tween containing 2% bovine non-fat dried milk protein (Sigma-Aldrich). Duplicate wells were incubated with plasma diluted 1:5 or with undiluted CSF. The human samples from the two age groups were pipetted on the plate in a randomized order. Serial dilutions of standards were prepared by using human recombinant alpha-synuclein in PBS/Tween. Beta-synuclein in PBS/Tween served as a negative control. After washing wells were incubated with polyclonal rabbit anti-alpha/beta/gamma-synuclein IgG (FL-140) sc-10717 (Santa Cruz Biotechnology, Santa Cruz, CA, USA) diluted 1:5000 in PBS/Tween for 1 h, followed by incubation with biotinylated goat F(ab’)2 anti-rabbit IgG (H+L chain specific) (Southern Biotech, AL, USA) diluted 1:5000 in PBS/Tween for 1 h. Streptavidin-POD enzyme conjugate (Roche, Mannheim, Germany) was used as an amplification step and tetramethylbenzidine (TMB) (Sigma-Aldrich) as the peroxidase substrate. The colour reaction was stopped after 30 minutes. Absorbance (OD, optical density) as a measure of specific human alpha-synuclein was measured at 450 nm in an ELISA reader (Sunrise, Tecan, Switzerland). All measured sample concentrations were corrected against the background control (sample blank, only plasma omitted) to calculate alpha-synuclein concentrations produced by the specific signal.

### Blocking of plasma alpha-synuclein

The specificity of measured plasma alpha-synuclein was demonstrated in a blocking assay by preincubation of plasma with monoclonal syn211 anti-alpha-synuclein antibody at 160 ng/ml for 2 h and 20°C prior to ELISA analysis. In addition, a solid phase alpha-synuclein preabsorption assay by means of a well to well plasma transfer was performed.

### Spike-and-recovery

To determine whether analyte detection is altered by sample matrix, i.e. plasma component, a known amount of alpha-synuclein was spiked into a plasma sample. If recovery is decreased, components of the sample matrix likely cause the difference. Spike-recovery assays were performed on plasmas from 11 individuals at the utilized dilution at 1:5.

### Acidification of plasma

Plasma was acidified to dissociate undetectable alpha-synuclein complexes with antibodies or chaperone-like proteins. Acidification was carried by methods adapted from Morgan et al. [19] after rigorous testing. In detail, 10 μl of plasma was diluted with 30 μl PBS/Tween buffer and acidified with 5 μl 1M glycine-acetate in PBS buffer, pH = 2.0, or control buffer, pH = 7.0, incubated at 20°C for 20 minutes in Eppendorf tubes on a shaker and subsequently neutralized with 5 μl Tris-HCl buffer, pH = 9.0. Final volume was 50 μl (plasma dilution of 1:5) and a neutral pH-value was ascertained using pH indicator strips (Sigma-Aldrich).

### SDS-PAGE and western blotting

Electrophoresis was performed with a 15% SDS polyacrylamide slab minigel and a Tris-glycine buffer system (SDS-PAGE). Human recombinant alpha-synuclein (600 ng) was loaded and subjected to 20 mA current until the bromophenol blue dye (Sigma-Aldrich) approached the end of the gel. Proteins were transferred at 150 mA for 4 h onto a 0.1 μm pore-size nitrocellulose membrane using a TRIS-glycine-methanol buffer. The membrane was washed in 0.1% Tween-20/PBS and blocked for 1 h with 5% skimmed milk (SM) (Sucofin, TSI GmbH & Co. KG, Zeven, Germany)/PBS. After washing, the membrane was cut into strips and incubated overnight at 4°C with 2% SM/PBS with 1:500 rabbit anti-alpha/beta/gamma-synuclein IgG (FL-140) or 1:1000 mouse syn211 anti-alpha-synuclein IgG antibody. After washing and blocking, strips were incubated with anti-rabbit or anti-mouse IgG-alkaline phosphatase (AP) at 1:5000 for 2 h in 2% SM/PBS. The immunoblot was visualized using BCIP (5-bromo-4-chloro-3-indolyl-phosphate) with NBT (nitro blue tetrazolium) (Promega, Mannheim, Germany) for detection of AP activity.

## Results

### Population and Demographics

Details of the population demographics are shown in Table 1. Age difference between the 80 healthy males was highly significant by unpaired two-tailed t-test, p<0.0001. Demographics of CSF samples were not analyzed because alpha-synuclein concentrations mostly fell below the detection limit of the assay (described below).

**Table 1:** Alpha-synuclein concentration in native and acidified plasma in a younger and older group of healthy males.

	**n**	**age (y)**	**alpha-synuclein native (ng/ml)**	**alpha-synuclein acidified (ng/ml)**
**young**	40	27.6 (26) +/- 0.7 CI 26.2–28.9	11.4 (4.3) +/- 3.2 CI 5.0–17.9	21.8 (9.7) +/- 4.8 CI 12.0–31.6
**old**	40	58.1 (58)+/- 0.7 CI 56.6–59.6	3.6 (1.5) +/- 1.4 CI 0.8–6.4	6.9 (3.6) +/- 2.6 CI 1.6–12.2

Data are shown as mean (median) +/- SE. SE = standard error, CI = 95% confidence interval of the mean, n = number of individuals, y = years.

### Measurement of human alpha-synuclein

**Fig 1:**
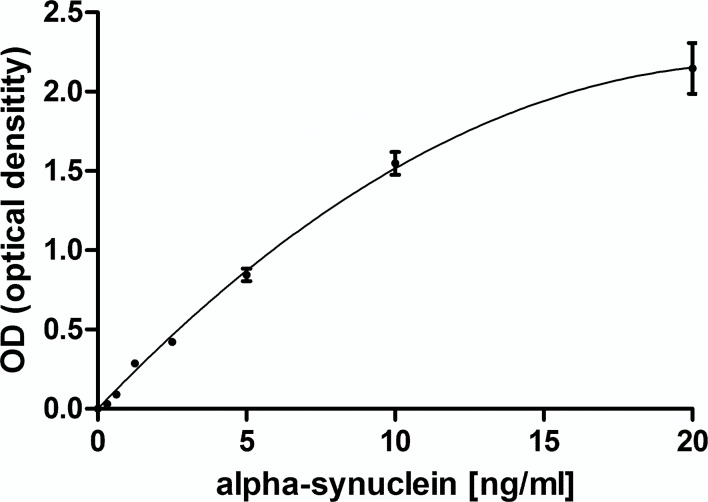
ELISA standard curve for alpha-synuclein. Detection limit of the assay was set at 300 pg/ml (described in results). SD bars are from duplicate measurements.

The ELISA was extensively tested with various modifications to achieve maximum specificity and reliability. After testing a wide range of plasma concentrations a dilution of 1:5 was chosen, because it provided alpha-synuclein concentrations generally within the linear range (0.3–10 ng/ml), well above the lower detection limit of the assay (300 pg/ml), which was 2-fold the unspecific background level given by a blank of 0.1 OD. Plasma samples below the detection limit were arbitrarily set to 100 pg/ml. The standard curve is illustrated in Fig 1. Measurements were replicated in 3 independent assays, the inter-assay coefficient of variation (%CV) measuring control plasma was 14%. In order to attain the highest consistency for a reliable comparison of alpha-synuclein concentrations, only data are shown that were generated within the same, last experimental run containing all samples.

### Stability of alpha-synuclein in standards and plasma

**Fig 2:**
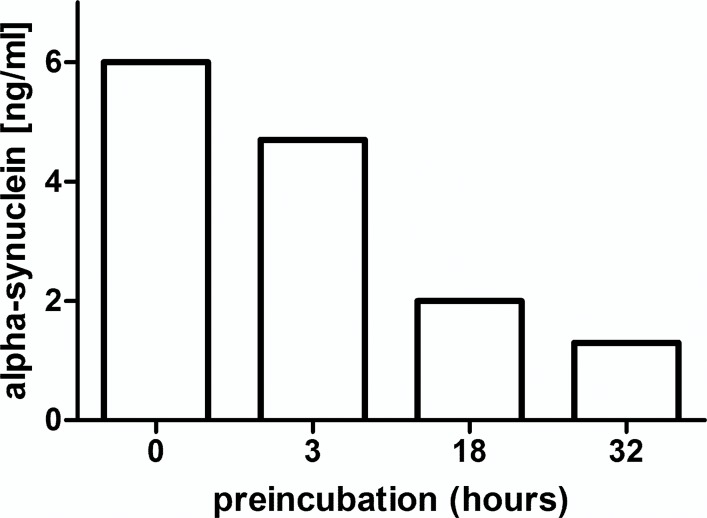
Concentration of an alpha-synuclein standard solution decreases with preincubation. Incubation of freshly diluted alpha-synuclein at 6 ng/ml in PBS for 3, 18 and 32 h at 37°C decreased ELISA-measured alpha-synuclein levels by 21, 66 and 78%, respectively.

**Fig 3:**
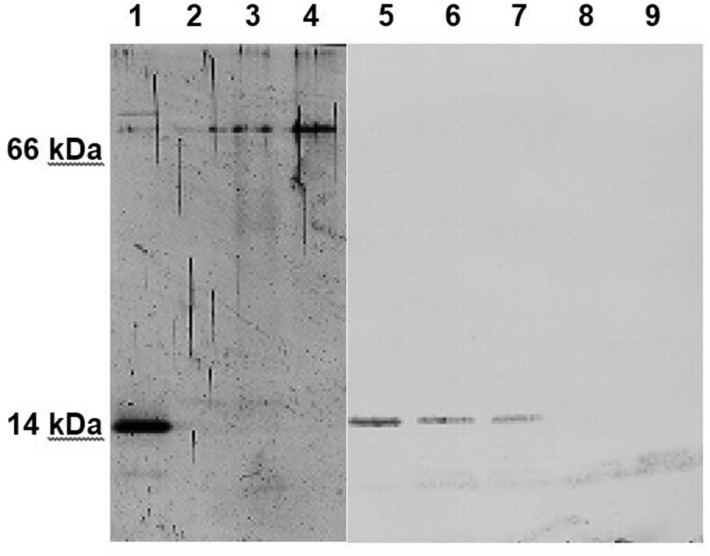
Western immunoblot of alpha-synuclein standard solution after preincubation. Lane 1–4 was probed with anti-alpha/beta/gamma-synuclein antibody (FL-140), lane 5–9 with anti-alpha/beta/gamma-synuclein antibody (syn211). Compared to freshly diluted alpha-synuclein in PBS at 6 ng/ml (0 h, lane 1 and lane 5), incubation at 37°C for 3, 18, 32 h (lane 2, 3, 4, respectively) resulted in fading of the monomeric alpha-synuclein band at 14 kDa and appearance of a higher molecular weight band using FL-140, whereas no higher molecular weight band was detected with syn211 after 1, 3, 5, 10 h (lane 6, 7, 8, 9, respectively).

We observed a diminution of the alpha-synuclein concentrations in standard solutions upon storage at room temperature. To investigate this observation in greater detail a 6 ng/ml alpha-synuclein standard solution was preincubated for various time periods before performing the assay. In parallel, western immunoblotting from same standards visualized novel conformations or aggregates. Compared to freshly thawed and diluted monomeric alpha-synuclein preincubation of equimolar concentrations of recombinant alpha-synuclein in PBS for 3, 18 and 32 h at 37°C resulted in a decrease of alpha-synuclein concentration by 21, 66 and 78%, respectively, as measured by ELISA (Fig 2) and visualized by western immunoblot (Fig 3). This was reflected by appearance of a higher molecular weight band at 70 kDa and time-dependent fading of the monomeric alpha-synuclein band at 14 kDa when probing with a polyclonal anti-alpha/beta/gamma-synuclein antibody (FL-140), lane 1–4 (Fig 3). A higher molecular weight band was not detected with monoclonal anti-alpha-synuclein antibody (syn211), lane 5–9 (Fig 3). No bands or degradation products could be observed below the alpha-synuclein band. In contrast to standard solution, alpha-synuclein concentrations in native plasma were more stable. Leaving undiluted plasmas at 20°C for 3, 24 or 48 h, resulted in an average decrease of 5, 13 and 17%, respectively. Preincubation of plasma at 37°C for 3 and 24 h lead to mean reduction of 5 and 16%, respectively. Furthermore, three freeze-thaw cycles of undiluted plasmas did not alter the alpha-synuclein concentrations significantly (data not shown).

### Specificity of alpha-synuclein measurements

**Fig 4:**
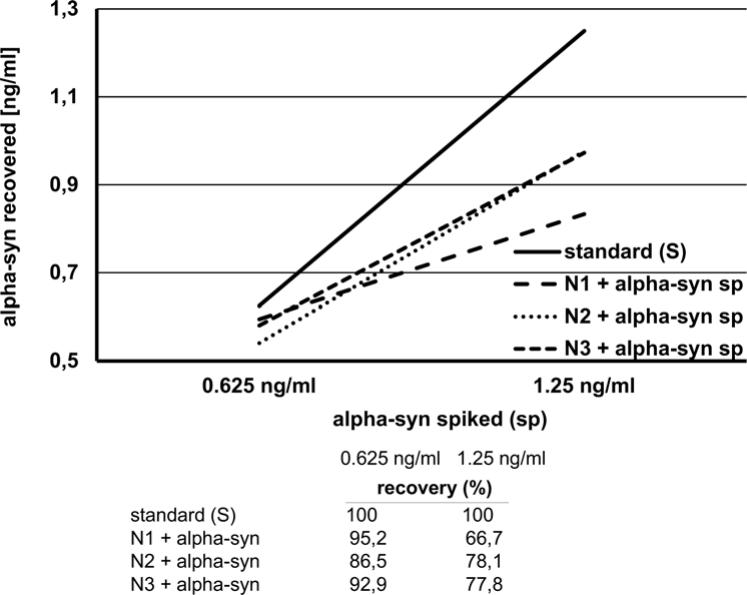
Spike and recovery. Three native plasmas N1, N2 and N3 were spiked (sp) with alpha-synuclein (alpha-syn) at 0.625 ng/ml and 1.25 ng/ml, equivalent to the amount that was added to standard solutions (S). Recovery (%) of spiked alpha-synuclein was calculated by subtraction of the internal alpha-synuclein in the native plasma (given in results).

**Fig 5:**
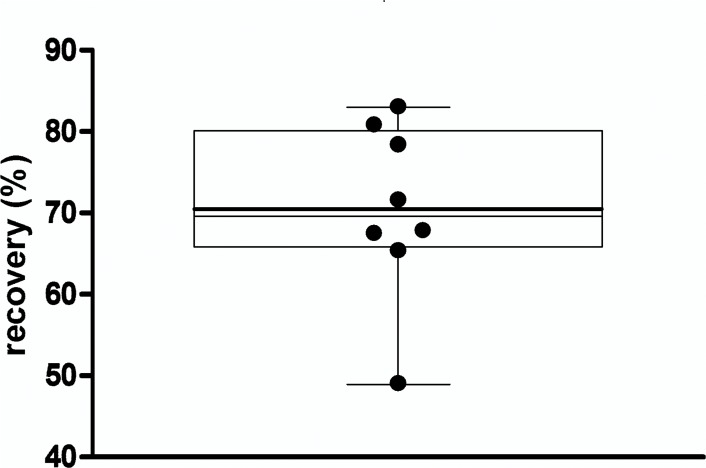
Spike and recovery. Eight native plasmas were spiked with 5 ng/ml alpha-synuclein, recoveries are illustrated in a box plot*, mean recovery was 70.4%.* The boxes upper and lower borders represent the 25th and 75th percentile, whiskers indicate the 10 to 90 percentile, lines within the box mark the median (thin line) and the mean (bold line).

To validate the specificity of plasma alpha-synuclein measurements, native plasma was preincubated with monoclonal syn211 anti-alpha-synuclein antibody to block the alpha-synuclein binding site. Syn211-blocked and untreated native plasma samples were incubated in parallel and subsequently measured by ELISA. Resulting alpha-synuclein concentrations were reduced by a mean of 71%, confirming the specificity of the measurements. Similarly, solid phase preabsorption of alpha-synuclein by a well-to-well transfer resulted in loss of the protein. Dilution of plasma yielded proportional decreases of alpha-synuclein concentrations (linearity-of-dilution). In spike-and-recovery assays three native plasma samples N1, N2 and N3 containing 0.23, <0.03 (below limit of assay) and 0.29 ng/ml alpha-synuclein, respectively, were spiked with 0.625 and 1.25 ng/ml synuclein, equivalent to the amount that was added to standard solutions (s) (Fig 4). Recovery (%) of spiked alpha-synuclein was calculated by subtraction of the internal plasma alpha-synuclein. Eight additional plasmas were spiked with 5 ng/ml alpha-synuclein, recovery is illustrated (Fig 5). Notably, recovery appeared to decline with increasing amounts of alpha-synuclein spiked into the plasma. When native unspiked plasmas N1 and N3 were blocked with syn-211, alpha-synuclein measurements diminished to 0.03 ng/ml and below the detection limit of the assay (<0.03 ng/ml), respectively. Finally, no specific signal or crossreactivity was observed when using recombinant β-synuclein (not shown).

### Plasma alpha-synuclein

Alpha-synuclein concentrations were measured in native plasma of 80 healthy control males, divided into a younger and older age group (Table 1). Plasma was acidified to dissociate alpha-synuclein-antibody or protein complexes and thus liberate masked alpha-synuclein for detection. Mean and median alpha-synuclein concentrations in native and acidified plasma are given in Table 1.

**Fig 6:**
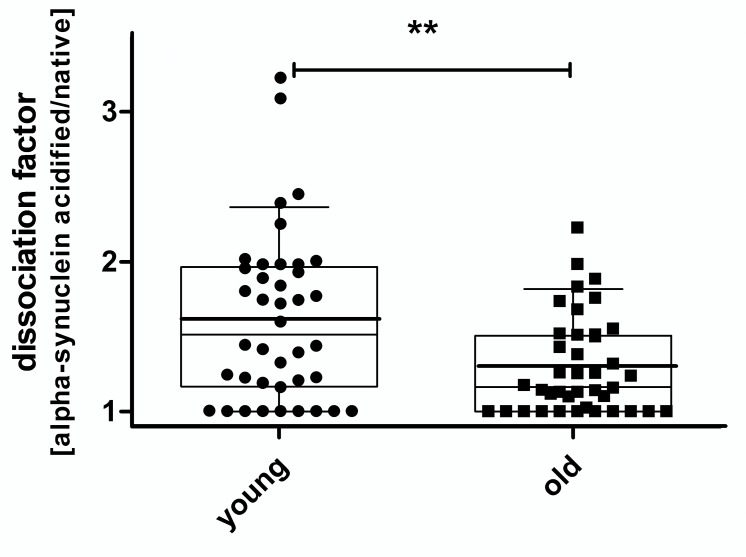
Dissociation factor after plasma acidification young vs. old. Box plots* of dissociation factors, calculated by dividing the acidified and native plasma alpha-synuclein concentration of each individual sample. Lower dissociation factors were observed in older individuals, very significant by Mann Whitney test, p = 0.0093.

Acidification resulted in higher alpha-synuclein concentrations in most plasmas. In detail, 30 out of 40 individuals (75%) in the younger group and 28 of 40 (70%) in the older group showed an increase. This was highly significant for both groups according to one-tailed Wilcoxon matched pairs test, p<0.0001. The increase of plasma alpha-synuclein levels was much stronger in the younger than older individuals. When calculating dissociation factors by dividing the acidified and native alpha-synuclein concentration from each individual lower multipliers were observed in older compared to younger individuals, very significant by Mann Whitney test, p = 0.0093 (Fig 6)

**Fig 7:**
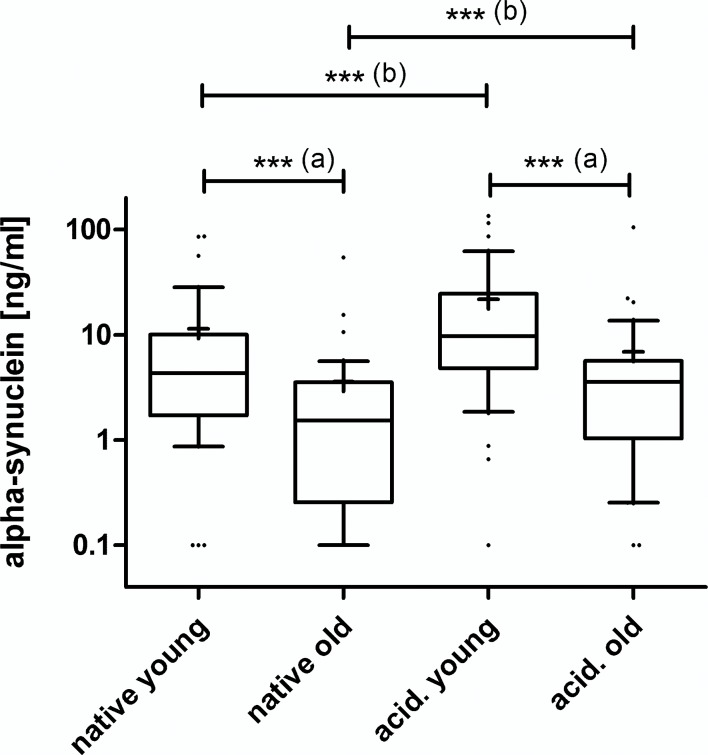
Box plots* of alpha-synuclein levels in native and acidified plasma. (a) Younger in comparison to older individuals revealed higher alpha-synuclein levels, highly significant by Mann Whitney test for native, p = 0,0005 and acidified plasmas, p<0.0001. (b) Plasma acidification increased alpha-synuclein concentrations in both age groups, highly significant by a one-tailed Wilcoxon matched pairs test, p<0.0001. The additional horizontal short line in the box represents the mean.

**Fig 8:**
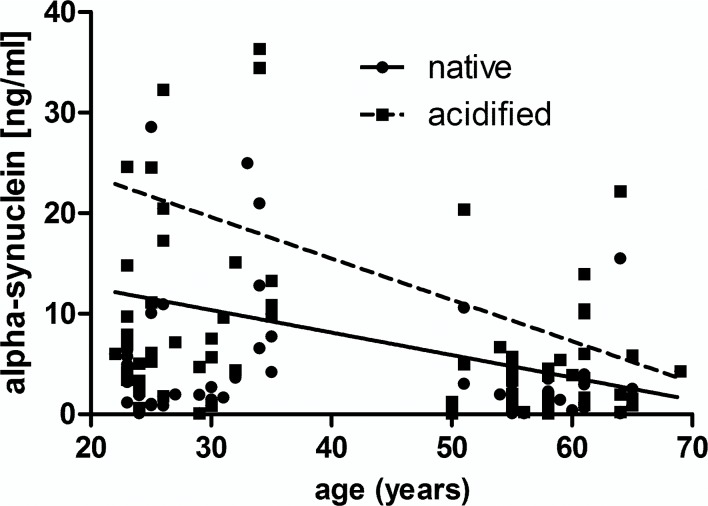
Linear regression of age with alpha-synuclein concentration in native and acidified plasma. Alpha-synuclein decreased with age and was enhanced by acidification. Spearman correlation was very significant for native samples, p = 0.0093, r = -0.3, after acidification, p = 0.0019, r = -0.34.

There were much higher alpha-synuclein concentrations in native plasma from younger compared to older individuals, highly significant by Mann Whitney test, p = 0.0005, shown in Fig 7. Remarkably, for acidified plasma the difference between the age groups was even more evident, p<0.0001. Eleven of 80 (14%) native and 3 of 80 (4%) acidified plasma samples were below the detection limit (300 pg/ml) of the assay. Ten of the total 14 (71%) negative plasma samples were from older individuals. In contrast, the alpha-synuclein concentration in CSF was approximately 10-folds lower than in plasma. Nine of 13 CSF samples (69%) were below the detection limit of the assay (300 ng/ml), and thus the results were unsuitable for further analysis. In Fig 8 the alpha-synuclein concentrations of native and acidified plasma are plotted against age for each individual, the respective linear regression line are indicated. Elevations between lines were significantly different, p = 0.039, and slopes differed significantly from zero with p = 0.046 and p = 0.021 for native and acidified plasma, respectively. The Spearman correlation was very significant for native samples, p = 0.0093, r = -0.3 and after acidification, p = 0.0019, r = -0.34. No correlation of alpha-synuclein concentrations with age was observed within the younger or older group, signifying falling alpha-synuclein plasma levels after the age of 35.

## Discussion

Prevention of age-related diseases is of pivotal interest to ascertain quality of life. To reach this goal, identification of aging biomarkers that reflect biological rather than chronological age, delineate tempo of aging and indicate impending life-span would be vital [10]. In addition, such biomarkers could potentially reveal dormant age-related diseases like movement and cognitive disorders and facilitate their primary prevention, reducing incidence and prevalence of disease. Thereby, compliance and sustained life-style changes could be achieved.

Aging is characterized by biological key events which involve protein homeostasis (proteostasis), DNA damage, telomere shortening, epigenetic alterations, mitochondrial dysfunction, caused by reactive oxygen species (ROS) or mitochondrial (mt)DNA mutations [20,21]. Based on evidence that protein aggregation constitutes an inherent phenomenon of aging [3,4] we explored alpha-synuclein plasma levels which may be altered in a dysfunctional proteostasis network, thought to influence life span already in early adulthood [5,6]. Homeostasis of alpha-synuclein may be disturbed early on by proteasomal and lysosomal malfunction that impair clearance of damaged or denatured proteins [22,23] or by lack of molecular chaperones or natural antibodies that protect or refold proteins [24–27]. Mutant and/or structurally altered alpha-synuclein may result in gain of toxic or loss of function by causing cell injury and death (i.e. oligomers or protofibrils) or by being probably inert (i.e. fibrils in Lewy bodies). Thus, maintaining protein function and appropriate tissue concentrations may improve healthy aging [28,29].

Alpha-synuclein was discovered in 1988 in CNS neurons [30] and cloned from AD brain tissue in 1993 [31]. In 1997 it was detected in hematopoietic cells [32], and as of 2000 in CSF and serum [33,34]. Subsequently, alpha-synuclein was explored as a biochemical marker in neurodegenerative diseases, particularly in PD as the prototypic synucleinopathy [35], reviewed in [36–39]. Alpha-synuclein is a principal component of Lewy bodies [40,41] the pathological hallmark of synucleinopathies [42]. In AD, a prototype tauopathy, up to 60% of patients have Lewy body pathology in the CNS [43,44]. In contrast to Abeta plaques, Lewy bodies and aggregates of alpha-synuclein are well correlated with cognitive impairment not only in PD but also in AD [45,46], signifying alpha-synuclein as a prime candidate biomarker. Hence, accurate quantification of alpha-synuclein in biological fluids is crucial, albeit complicated due to low concentrations and its tendency to self-aggregate and adhere to plastic surfaces [47]. We observed diminishing alpha-synuclein levels after in vitro aggregation of standard solutions, which has also been observed in ELISAs measuring Abeta [48]. In comparison, leaving undiluted plasma for prolonged time at room temperature led to a modest reduction in the alpha-synuclein concentrations. Accordingly, standard operating procedures for sample handling and assay conditions should be defined; a unified effort to standardize measurements to improve reproducibility is ongoing [49].

Many studies have addressed the analysis of alpha-synuclein in CSF [36,37] and to a lesser extent in blood plasma or serum [50–62]. Available data is conflicting and plasma or serum concentrations vary, mostly between 4–48 ng/ml. No consistent differences between patients and controls or within healthy populations could be established. We detected low alpha-synuclein plasma levels of 3.6 ng/ml (mean) in healthy older males (mean 58 years). Alpha-synuclein negatively correlated with age showing significantly higher concentrations of 11.4 ng/ml (mean) in younger individuals (mean 28 years). Plasma acidification served to enhance this difference which may reflect a decrease in alpha-synuclein-antibody complexes or chaperone activity in the elderly. To our knowledge similar observations have not been reported previously and could be related to the inclusion of younger individuals in addition to methodological differences. Our assay was rigorously validated and background levels were very low. Interestingly, Brighina et al. observed a positive correlation of alpha-synuclein levels with age in lymphomonocytes from healthy controls [63], in accordance with the notion that intracellular accumulation of alpha-synuclein could lead to lower plasma levels.

The above concept that alpha-synuclein may be complexed with plasma components such as antibodies or other binding proteins which are liberated by acidification is supported by loss of recovery after spiking alpha-synuclein into the plasma sample. Because higher molecular weight aggregates of alpha-synuclein were not detected in the western immunoblot when probing with coating antibody (syn211), it is reasonable to assume that the detecting epitope can be conformationally masked in aggregates, which then remain undetected in the assay. This may partly explain the decrease of alpha-synuclein levels after prolonged incubation of plasma or standard solution.

Body homeostasis is characterized by a subtle equilibrium of de- and regeneration, renewal and removal of damaged cells and proteins. Protein turnover is critically reduced in later life [64] resulting in catabolism and accumulation of damaged proteins in muscle and brain [65]. Already at the end of the second life decade this balance may become instable and skewed towards senescence and degeneration. As such, elite level performance in sports will top out at 24–28 years of age, an age range represented by our younger healthy males, and declines thereafter [66,67]

Alpha-synuclein may have broad implications for healthy aging because it presumably plays a key role in synaptic plasticity [68,69]. It is critical for memory formation and lifelong learning that declines in healthy humans within age-adjusted norms, also known as age-related memory impairment, but is only disrupted in neurodegenerative disease. Also, alpha-synuclein may trigger transient or bland symptoms of unknown significance that may occur in healthy people or precede disease by many years or possibly even decades. Interestingly, animals do not spontaneously develop PD and complete loss of alpha-synuclein in knock-out mice does not cause overt disease, except for deficits in the dopamine system of the basal ganglia [70]. These mice show normal development and, surprisingly, resistance to mitochondrial toxins such as MTPT [71].

The potential relevance of alpha-synuclein as an aging biomarker is further supported by its characteristic to signify a broad spectrum of neurodegenerative diseases, presenting with variable neurological, psychiatric or somatic symptoms caused by more or less widespread alpha-synuclein accumulation or deposition in the CNS and PNS. Clinical symptoms may include extrapyramidal signs (EPS), cognitive impairment, dementia, affective disorders, psychosis and autonomic symptoms, i.e. sleep disturbances and incontinence. In addition, it is conceivable that function of other tissues and cell types such as leukocytes that express alpha-synuclein may be altered [32,56,72]. Indeed, recent work revealed that macrophages/microglia expressing alpha-synuclein at higher levels exhibit impaired cytokine production and immune function [73]. Similar to Abeta, alpha-synuclein forms toxic soluble intermediates, such as oligomers or protofibrils [74] when reaching critical concentrations that likely play pathophysiological key roles in a diverse spectrum of neurodegenerative diseases. In this context, it is of note that alpha-synuclein gene multiplications or missense mutations raise alpha-synuclein levels in blood and brain and lead to early onset and severe forms of PD or DLB, demonstrating that gene dosage alone causes variable disease phenotypes [75–79].

In summary, our data reveal that alpha-synuclein levels in blood plasma from healthy individuals markedly decrease between the 3^rd^ and 5^th^ decade of life. This disturbed proteostasis may relate to the frailty process but not a particular disease, possibly caused by dysregulation of cellular lysosomes and proteasomes, antibodies or molecular chaperones that modify alpha-synuclein levels and conformation, respectively. Thus, alpha-synuclein may reflect biological age and qualify as a candidate biomarker of aging to advance life expectancy. Moreover, our findings support the idea that imbalances of alpha-synuclein levels may influence neuronal vulnerability that increases with age, a main risk factor for neurodegenerative disease [80].
